# Investigation of Lead and Chromium Exposure After Consumption of Contaminated Cinnamon-Containing Applesauce — United States, November 2023–April 2024

**DOI:** 10.15585/mmwr.mm7414a2

**Published:** 2025-04-24

**Authors:** Alyssa N. Troeschel, Melanie C. Buser, Andrea Winquist, Perri Ruckart, Michael Yeh, David Kuai, Arthur Chang, Audrey F. Pennington, Jelonia T. Rumph, Madison R. Smith, Marisol Valenzuela Lara, Natalie Cataldo, Kailey Lewis, Katherine Arnold, Stic Harris, David C. Nicholas, Megan Hughes, Teresa Wortmann, Ed Norman, Melanie D. Napier, Jamaica Dillard, Johnni Daniel

**Affiliations:** ^1^Division of Environmental Health Science and Practice, National Center for Environmental Health, CDC; ^2^Geospatial Research, Analysis, and Services Program, Agency for Toxic Substances and Disease Registry, CDC; ^3^Human Foods Program, Food and Drug Administration, College Park, Maryland; ^4^New York State Department of Health; ^5^Department of Epidemiology & Biostatistics, University at Albany, State University of New York, Rensselaer, New York; ^6^Missouri Department of Health and Senior Services; ^7^North Carolina Department of Health and Human Services, Division of Public Health; ^8^Arizona Department of Health Services.

**Summary**

**What is already known about this topic?**

In October 2023, the Food and Drug Administration was alerted to several cases of lead poisoning linked to consumption of applesauce containing cinnamon contaminated with lead and chromium.

**What is added by this report?**

In November 2023, CDC initiated a national call for cases and identified 566 cases of lead poisoning after consumption of cinnamon-containing applesauce in 44 U.S. states, the District of Columbia, and Puerto Rico. Symptoms potentially consistent with lead poisoning were reported in temporal association with consumption of the applesauce in approximately 20% of cases.

**What are the implications for public health practice?**

This incident highlights the importance of preventing toxic metal contamination of food; childhood blood lead testing and follow-up to identify lead poisoning events; and educating clinicians and public health practitioners about the potential for toxic metal exposure from less well-known sources, including food.

## Abstract

Although lead poisoning can cause detrimental health effects, it is largely preventable. Common exposure sources include contaminated soil, water, and lead-based paint in homes built before the 1978 ban on residential lead-containing paint. In North Carolina, testing for lead is encouraged for all children at ages 1 and 2 years, and is required for children covered by Medicaid. In October 2023, routine pediatric blood lead testing and follow-up investigations conducted by the North Carolina Department of Health and Human Services identified four asymptomatic cases of lead poisoning associated with consumption of cinnamon-containing applesauce packaged in pouches. The Food and Drug Administration (FDA) identified lead in the cinnamon as the source of contamination; chromium was later also detected in the cinnamon. FDA alerted the public on October 28, and the distributor initiated a voluntary recall the following day. To estimate the impact of the event and characterize reported cases, CDC initiated a national call for cases (defined as a blood lead level [BLL] ≥3.5 *μ*g/dL in a person of any age in ≤3 months after consuming a recalled cinnamon-containing applesauce product). During November 22, 2023–April 12, 2024, a total of 44 U.S. states, the District of Columbia, and Puerto Rico reported 566 cases (55% in children aged <2 years, including 20% that were temporally associated with symptoms). The median maximum venous BLL was 7.2 *μ*g/dL (range = 3.5–39.3 *μ*g/dL). The hundreds of children poisoned by this incident highlight the importance of preventing toxic metal contamination of food and promoting routine childhood blood lead testing and follow-up to identify lead exposure sources. Clinicians and public health practitioners should be aware of the potential for exposure to toxic metals from less common sources, including food.

## Introduction

In October 2023, the North Carolina Department of Health and Human Services (NCDHHS) and North Carolina Department of Agriculture and Consumer Services notified the Food and Drug Administration (FDA) of an investigation into lead poisoning identified in four children aged 1–3 years, linked to consumption of cinnamon-containing applesauce (*1*). All cases were identified through routine childhood lead surveillance that detected a blood lead level (BLL) ≥5 *μ*g/dL, resulting in the eligibility of those children for a home lead source investigation (*1*). In North Carolina, blood lead testing is encouraged for all children at ages 1 and 2 years and is required for those covered by Medicaid (*1*). Although lead testing policies differ by state (*2*), NCDHHS conducts home investigations to identify sources of lead exposure when children aged <6 years have two consecutive capillary or venous BLLs ≥5 *μ*g/dL within a 12-month period (*1*). All four affected children were found to have consumed the same brand of cinnamon-containing applesauce. Laboratory testing found lead concentrations in pouches obtained from the affected children’s homes ranging from 1.9 to 3.0 parts per million (*1*), nearly 200–300 times the recommended action level for fruit purees and similar products intended for babies and young children (*3*).

FDA issued a public health alert on October 28, and the distributor of the contaminated applesauce initiated a voluntary recall the following day (*4*). Six days later, on November 3, after learning that the product that was sold under the WanaBana brand was also sold under two additional brand names (Schnucks and Weis), FDA expanded the alert to include the additional brands; the distributor later expanded the recall to include 2,998,088 pouches. Because of the known toxic effects of lead exposure on multiple organ systems and associated effects on neurodevelopment (*5*), CDC initiated a national call for cases of lead poisoning associated with consumption of the recalled cinnamon-containing applesauce to estimate the number of persons affected by this contamination event and characterize reported cases. This report summarizes the findings of that investigation.

## Methods

### Case Ascertainment and Data Collection

On November 21, 2023, CDC issued an alert^†^ requesting that states collaborate with their childhood lead poisoning prevention program to compile and report all cases of lead poisoning using a standard case definition. A case was defined as a BLL ≥3.5 *μ*g/dL in a blood sample obtained ≤3 months after consumption of a recalled cinnamon-containing applesauce product. CDC requested that states classify cases as suspected, probable, or confirmed, based on type of testing, completion of a follow-up environmental assessment, and identification of other potential sources of lead exposure (Box). CDC uses a blood lead reference value of 3.5 *μ*g/dL to identify children with BLLs higher than most children (i.e., in the top 2.5%) (*6*). The reference value is based on the 97.5th percentile of BLLs among U.S. children aged 1–5 years from the National Health and Nutrition Examination Survey cycles 2015–2016 and 2017–2018. States might use different BLLs to trigger public health action (e.g., follow-up environmental assessments) (*2*).

BOX **Case status classifications used in CDC’s national call for cases of lead poisoning associated with the consumption of recalled cinnamon-containing applesauce pouches — United States, November 2023–April 2024**

**Suspected**

BLL of ≥3.5 *μ*g/dL* detected through capillary or unspecified testing (not yet confirmed through venous blood testing) ≤3 months after consuming a recalled product^†^ and after November 2022.^§^

**Probable**

BLL of ≥3.5 *μ*g/dL* detected through venous testing ≤3 months after consuming a recalled product^†^ and after November 2022^§^ and one of the following:

- a follow-up environmental assessment to rule out other potential sources of lead exposure was not completed; or
- a follow-up environmental assessment was completed, but the results indicated other potential sources of lead exposure (e.g., lead-based paint)

**Confirmed**

BLL of ≥3.5 *μ*g/dL* detected through venous testing ≤3 months after consumption of a recalled product^†^ after November 2022^§^ and

- a follow-up environmental assessment to determine potential sources of lead exposure was completed; and
- the environmental assessment results indicated no other significant sources of lead exposure

**Abbreviation**: BLL = blood lead level.

* CDC uses a blood lead reference value of 3.5 *μ*g/dL to identify children with BLLs that are higher than most children’s levels. This level is based on the 97.5th percentile of the blood lead values among U.S. children aged 1–5 years from the 2015–2016 and 2017–2018 National Health and Nutrition Examination Survey cycles. Children with BLLs at or above the blood lead reference value are among the 2.5% of U.S. children with the highest BLLs.

^†^ A list of recalled products can be found on the Food and Drug Administration website https://www.fda.gov/food/outbreaks-foodborne-illness/investigation-elevated-lead-chromium-levels-cinnamon-applesauce-pouches-november-2023 and at https://www.fda.gov/safety/recalls-market-withdrawals-safety-alerts/wanabana-recalls-wanabana-weis-and-schnucks-apple-cinnamon-fruit-puree-pouches-cinnamon-apple-sauce.

^§^ According to the Food and Drug Administration, the recalled products were first sold in November 2022.

CDC provided states with a template for collecting information, including the case classification; demographic characteristics; estimated first and last dates of product consumption; BLL test type, date, and result; whether an environmental assessment or home investigation was conducted, and if so, investigation results; reported symptoms^§^ (Supplementary Box, https://stacks.cdc.gov/view/cdc/177614#tabs-3); and any hospitalizations. States submitted reports biweekly to CDC through a secure website during November 21, 2023–April 12, 2024.

### Analysis

After the reporting period, consistency in application of the case definition was ascertained by verifying that the reported information, including the BLL result, BLL test type, whether an environmental assessment was conducted, and the environmental assessment result, aligned with the reported case status. For example, all suspected cases were reviewed to verify that BLLs ≥3.5 *μ*g/dL were detected through capillary (rather than venous) testing. Confirmed cases were reviewed to verify BLLs ≥3.5 *μ*g/dL were detected through venous testing and that an environmental assessment that indicated no other significant sources of lead exposure had been completed. If submitted information did not align with reported case status, CDC contacted states for clarification and, if necessary, reclassified case status. Because information about product consumption dates was incomplete, whether the BLL test was conducted within the appropriate timeframe (i.e., in ≤3 months of consuming a recalled product) was not included in the verification process.

Multiple BLL results could be reported for any person. For this analysis, the maximum venous BLL reported for a given patient was recorded; this maximum value was summarized across all cases to obtain the median value (referred to as the median within-person maximum) and range. Data were summarized using SAS (version 9.4; SAS Institute). This activity was reviewed by CDC, deemed not research, and was conducted consistent with applicable federal law and CDC policy.^¶^

## Results

### Cases of Lead Poisoning Associated with Consumption of Contaminated Products

As of April 12, 2024, a total of 44 states, the District of Columbia, and Puerto Rico reported 566 cases to CDC (Figure); the largest numbers of cases were reported from New York (65), Missouri (50) and Arizona (38); these states accounted for 27% of all cases reported. Overall, 130 (23%) confirmed, 401 (71%) probable, and 35 (6%) suspected cases were reported (Table).

**FIGURE:**
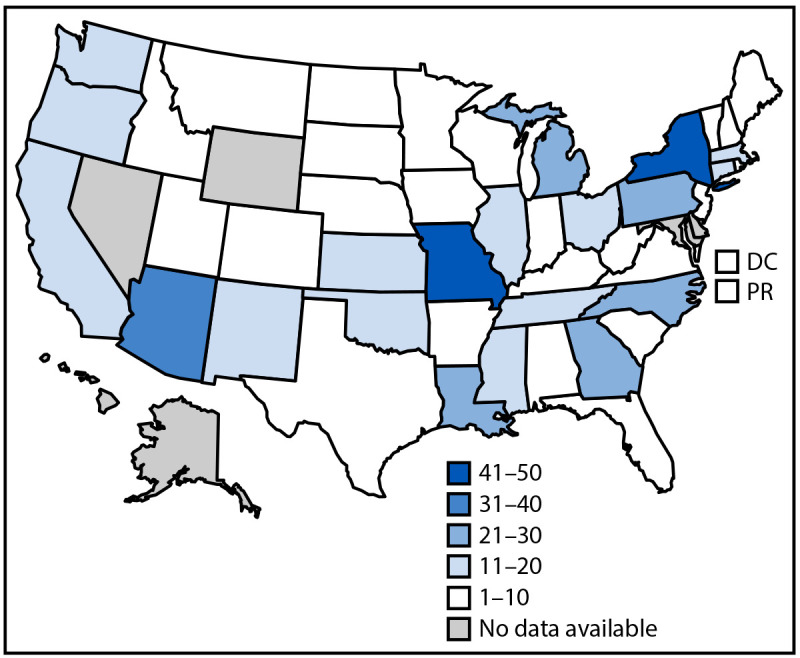
Reported number of cases* of lead poisoning associated with consumption of recalled cinnamon-containing applesauce products packaged in pouches, by jurisdiction^†^ (N = 566) — United States, November 2023–April 2024 **Abbreviations**: BLL = blood lead level; DC = District of Columbia; PR = Puerto Rico. * Includes the total number of confirmed, probable, and suspected cases. At a minimum, cases were reported to have a BLL ≥3.5 *μ*g/dL detected through venous, capillary, or unspecified testing ≤3 months after consuming a recalled WanaBana, Schnucks, or Weis brand fruit puree product after November 2022. ^†^ Some states have a routine threshold for investigation of BLLs higher than CDC’s blood lead reference value of 3.5 *µ*g/dL and would not have done routine investigations for some potential cases before the call for cases. States also had differences in resources available for investigating and reporting cases (especially cases that occurred before the recall in October 2023). In addition, guidelines for routine blood lead testing for children not enrolled in Medicaid and policies encouraging blood lead testing vary by state, with some states recommending universal screening and others recommending targeted screening. For these reasons, the distribution of the number of reported cases across states does not necessarily reflect the true public health impact of the contamination event across states.

**TABLE:** Characteristics* of persons with lead poisoning associated with the consumption of recalled cinnamon-containing applesauce packaged in pouches reported to CDC (N = 566) — United States, November 2023–April 2024

Case status	No. (%)
Confirmed^†^	130 (23.0)
Probable^§^	401 (70.8)
Suspected^¶^	35 (6.2)
**Age group, yrs**	
<2	311 (55.2)
2–5	231 (41.0)
≥6	21 (3.7)
**Sex at birth**	
Female	253 (47.6)
Male	278 (52.4)
**Race and ethnicity**	
Black or African American, non-Hispanic	76 (19.9)
White, non-Hispanic	216 (56.7)
Hispanic or Latino, any race	64 (16.8)
Other, non-Hispanic	25 (6.6)
Maximum venous BLL (*μ*g/dL) ≤3 months after consumption of a recalled product after November 2022 (among 531 probable and confirmed cases)**	
Mean (SD)	9.2 (5.85)
Median	7.2
Range	3.5–39.3
IQR	5.2–11.3
**Range of maximum venous BLL (*μ*g/dL) ≤3 months after consumption of a recalled product after November 2022 (among 531 probable and confirmed cases)**	
3.5 to <5	103 (19.4)
5 to <10	260 (49.0)
10 to <15	98 (18.5)
15 to <20	34 (6.4)
20 to <25	20 (3.8)
25 to <30	11 (2.1)
30 to <35	3 (0.6)
≥35	2 (0.4)
**Date of first consumption (n = 341)**	
Median	Jun 1, 2023
Range	Nov 1, 2022–Jan 1, 2024
**Date of last consumption (n = 458)**	
Median	Oct 31, 2023
Range	Dec 1, 2022–Mar 31, 2024
**Date of first BLL ≥3.5** ***μ*g/dL ≤3 months after consumption of a recalled product after November 2022**^††^	
Median	Oct 20, 2023
Range	Nov 2, 2022–Mar 28, 2024
**Environmental assessment conducted**	245 (44.1)
**Signs or symptoms reported**	
Any reported sign or symptom	81 (20.0)
Any gastrointestinal sign or symptom^§§^	55 (13.6)
Abdominal pain^§§^	17 (4.2)
Constipation^§§^	15 (3.7)
Vomiting/Nausea^§§^	16 (4.0)
Diarrhea^§§^	10 (2.5)
Other gastrointestinal sign or symptom^§§^	21 (5.2)
Any developmental or behavioral sign or symptom^§§^	35 (8.6)
Altered mood or behavior^§§^	23 (5.7)
Developmental delay sign or symptom^§§^	18 (4.4)
Lethargy/Fatigue^§§^	17 (4.2)
Headaches^§§^	10 (2.5)
Other signs or symptoms^§§,¶¶^	22 (5.4)

**Abbreviations:** BLL = blood lead level; SD = standard deviation.

* The following variables had missing data: age (three); sex (35); race and ethnicity (185); maximum venous BLL (35); date of first consumption (225); date of last consumption (108); environmental assessment (10); and reported signs or symptoms (161).

^†^ A person with a confirmed case status had to meet each of the following criteria: 1) BLL of ≥3.5 *μ*g/dL confirmed through venous testing ≤3 months after consuming a recalled WanaBana, Schnucks, or Weis brand fruit puree product after November 2022 and 2) a follow-up environmental assessment indicated no other significant sources of lead exposure.

^§^ A person with a probable case status had to have a BLL of ≥3.5 *μ*g/dL confirmed through venous testing ≤3 months after consuming a recalled WanaBana, Schnucks, or Weis brand fruit puree product after November 2022 and had to meet one of the following two criteria: 1) a follow-up environmental assessment was not conducted or 2) a follow-up environmental assessment was conducted, but the environmental assessment results indicated other likely potential sources of lead exposure.

^¶^ A person with a suspected case had to have a BLL of ≥3.5 *μ*g/dL detected through capillary or unspecified testing, not yet confirmed via venous blood testing, ≤3 months after consuming a recalled WanaBana, Schnucks, or Weis brand fruit puree product after November 2022.

** The maximum reported BLL measured through venous testing ≤3 months after consumption of a recalled product after November 2022 was calculated for each person with a reported case (potentially among several reported BLL results). The maximum reported venous lead level was missing for persons who only received capillary tests.

^††^ The earliest date of a blood lead test exceeding the threshold was the earliest date on which the person had a blood lead test result of ≥3.5 *μ*g/dL (either capillary or venous) in ≤3 months of consuming a recalled product after November 2022.

^§§^ Among those for whom symptom information was available (not missing). Signs or symptoms were reported in a single open-text field. Persons could report multiple signs or symptoms. Reported signs or symptoms were reviewed and categorized.

^¶¶^ Other reported signs or symptoms included anemia (one), canker sores (one), fever (three), unspecified cramps (one), muscle spasms (one), crossed eyes (one), white stools (one), weight loss (six), difficulty sleeping (four), failure to thrive (two), balance problems (one), pain (one), and muscle or joint pain (one).

### Characteristics of Cases

Children aged <6 years accounted for 542 (96%) cases, 311 (55%) of which were in children aged <2 years. Among probable and confirmed cases, the median within-person maximum venous BLL was 7.2 *μ*g/dL (range = 3.5–39.3 *μ*g/dL; IQR = 5.2–11.3 *μ*g/dL); approximately one third (32%) of values were ≥10 *μ*g/dL. Estimated date ranges during which persons first and last consumed a recalled product were November 1, 2022–January 1, 2024, and December 1, 2022–March 31, 2024, respectively. No hospitalizations were reported. Clinical signs and symptoms were reported in temporal association with 81 cases (approximately 20%) including 55 that were gastrointestinal, and 35 that were developmental or behavioral.

## Public Health Response

On November 3, 2023, CDC notified its funded Childhood Lead Poisoning Prevention Programs (CLPPPs)** about the recall and on November 13, issued an alert to clinicians via the CDC Health Alert Network (Supplementary Figure, https://stacks.cdc.gov/view/cdc/177614#tabs-3). On December 5, CDC published a website including recommendations for the public and businesses, which was updated biweekly with case counts. On January 5, 2024, FDA reported recalled products and cinnamon collected from the foreign manufacturer contained high levels of both lead and chromium (*4*). CDC then issued a Clinician Outreach and Community Activity (COCA) Now (https://www.cdc.gov/coca/hcp/about/index.html) report, alerting clinicians about the possibility of chromium exposure. On February 29, 2024, after additional analysis of the cinnamon, FDA reported that the lead and chromium previously detected in the cinnamon were from lead chromate.

## Discussion

This investigation identified 566 cases of high BLLs after consumption of applesauce containing cinnamon that was contaminated with lead and chromium. Reported signs and symptoms included abdominal pain, lethargy, and developmental or behavioral symptoms, all of which, although nonspecific, are consistent with lead poisoning (*5*). The long-term implications of this event remain unknown.

Lead can affect nearly every organ system, with central nervous system effects being the primary concern in pediatric exposures, causing harm that might not be immediately apparent and that might be lifelong (*5*). The presence of lead in blood is associated with declines in cognitive and neuromotor and neurosensory function. Although exact levels at which this occurs in acute poisoning events is unclear (*5*), there is no safe BLL in children (*5*). Health effects of eating food contaminated with chromium (VI) (hexavalent chromium), the form found in lead chromate, are not well understood. Most data on health effects of hexavalent chromium come from inhalational and dermal exposures in the workplace, where long-term exposures have resulted in lung disease, ulceration of mucous membranes, and cancer (*7*). CDC did not recommend for or against testing chromium levels in blood, serum, or urine among persons affected by this event. Chromium levels only reflect recent exposures, because chromium is rapidly eliminated from the body. Results are difficult to interpret and do not guide clinical management, because the levels at which chromium causes harm are not well established. CDC did recommend blood lead testing for children who consumed a recalled product because results can guide follow-up actions for prevention and medical treatment.

This investigation highlights the importance of primary prevention, i.e., preventing toxic exposures before they occur. Increased product testing is essential to identifying contaminated products and preventing them from entering the food supply. However, the globalized food and consumer good supply chain might increase the potential for outbreaks of lead poisoning and underscores the continued need for childhood blood lead testing and resources to support follow-up investigations and referrals for services to protect health. CDC’s CLPPP has helped build capacity for state and local childhood lead poisoning prevention programs, which were essential throughout this investigation. In North Carolina, where the first cases were identified, CDC had provided nearly three decades of support to CLPPP and the state has a cadre of highly experienced and skilled public health professionals who were able to quickly identify and act on those cases.

The most common sources of lead exposure include lead-contaminated paint, soil, and water; however, this investigation highlights the importance of considering lesser known sources of toxic metal exposures. FDA determined that cinnamon ground in Ecuador was the source of the contamination, through finished product testing and testing of the cinnamon ingredient. FDA analyzed ground cinnamon samples and found lead chromate, using an internally validated method. Although the source of lead chromate is unknown, the presence of this compound is indicative of economic adulteration (*4*). Economically motivated adulteration, also known as food fraud, is designed to avoid detection. Adulteration of spices, such as paprika and turmeric, with lead chromate and other lead-containing compounds to enhance weight and color has been reported (*8*–*10*). Lead has also been found in candies and nonfood products imported from other countries, such as ceramic and glassware products, cosmetics, and traditional medicines.^††^

### Limitations

The findings in this report are subject to at least six limitations. First, cases were almost certainly underreported, with the degree of underreporting likely varying by time (e.g., persons who last consumed the product before the recall in late October 2023 were more likely to be underreported), age (e.g., persons aged ≥2 years are less likely to undergo routine BLL testing)^§§^ (*2*), and state (e.g., guidelines for routine blood lead testing for children not enrolled in Medicaid and policies encouraging blood lead testing vary by state, with some states recommending universal testing and others recommending targeted testing) (*2*). Second, data on the amount of product consumed were not collected in a standardized way. Third, reported signs and symptoms were collected through an open-text field that was often left blank. Fourth, dates of first or last consumption were often left blank, possibly as a result of difficulty in remembering the date of last consumption. Fifth, although CDC verified whether submitted data aligned with reported case classification for most elements, verifying whether BLL tests were conducted within the appropriate time frame was not possible because a high percentage of consumption dates were missing. Finally, additional sources of lead exposure contributing to cases cannot be completely excluded (i.e., some persons might have had BLLs ≥3.5 *μ*g/dL and consumed a recalled product in ≤3 months but had lead exposure from another source, such as paint).

### Implications for Public Health Practice

This investigation found that at least 542 children aged <6 years had high BLLs after consuming applesauce containing lead- and chromium-contaminated cinnamon. Primary prevention (i.e., stopping toxic metal exposure before it occurs) remains the best way to prevent lead and chromium poisoning, though childhood blood lead testing and follow-up remain critical secondary prevention strategies when primary lead prevention efforts fail. This national lead poisoning outbreak from an adulterated spice underscores the importance of clinicians and public health practitioners maintaining a high index of suspicion for toxic metal exposures from lesser-known sources. This investigation highlights the importance of coordination among CDC, FDA, and state public health partners in responding to a large-scale foodborne outbreak, leading to the recall of a nationally distributed lead- and chromium-contaminated food product marketed primarily to young children.

## Footnotes

* These senior authors contributed equally to this report.

† CDC issued this request through the Epidemic Information Exchange.

§ According to FDA, the recalled products were first sold starting in November 2022, and states collected data about symptoms during November 2023–April 2024. Therefore, reported symptoms include those experienced in the shorter term and do not reflect potential longer-term effects.

¶ 45 C.F.R. part 46.102(l)(2), 21 C.F.R. part 56; 42 U.S.C. Sect. 241(d); 5 U.S.C. Sect. 552a; 44 U.S.C. Sect. 3501 et seq.

** CLPPP is a CDC-funded program dedicated to eliminating childhood lead poisoning. There are currently 62 state and local childhood lead poisoning prevention programs are in operation (representing 47 U.S. states, the District of Columbia, Puerto Rico, and 13 localities). CLPPP was an ideal mechanism through which to communicate with public health professionals involved in investigating cases of lead poisoning.

††
https://www.cdc.gov/lead-prevention/prevention/foods-cosmetics-medicines.html

§§ Routine lead testing is typically done for children aged <72 months at high risk, based on age of housing and sociodemographic risk factors (most commonly at age 12–24 months; blood lead testing is required for all children enrolled in Medicaid at ages 12 and 24 months) and for adults at risk for occupational lead exposure.
